# Development and validation of a prognostic nomogram in gastric cancer with hepatitis B virus infection

**DOI:** 10.1186/s12967-019-1841-3

**Published:** 2019-03-25

**Authors:** Yi He, Minjie Mao, Wenjuan Shi, Zhonglian He, Lin Zhang, Xueping Wang

**Affiliations:** 1 Department of Laboratory Medicine, State Key Laboratory of Oncology in South China, Collaborative Innovation Center for Cancer Medicine, Sun Yat-sen University Cancer Center, Guangzhou, China; 2 Department of Information Section, State Key Laboratory of Oncology in South China, Collaborative Innovation Center for Cancer Medicine, Sun Yat-sen University Cancer Center, Guangzhou, China

**Keywords:** Gastric cancer, Prognosis, Nomogram, Liver function

## Abstract

**Background:**

Patients with HBsAg-positive gastric cancer (GC) are a heterogeneous group, and it is not possible to accurately predict the overall survival (OS) in these patients.

**Methods:**

We developed and validated a nomogram to help improve prediction of OS in patients with HBsAg-positive GC. The nomogram was established by a development cohort (n = 245), and the validation cohort included 84 patients. Factors in the nomogram were identified by univariate and multivariate Cox hazard analysis. We tested the accuracy of the nomograms by discrimination and calibration, and plotted decision curves to assess the benefits of nomogram-assisted decisions in a clinical context. Then we evaluated the risk in the two cohort.

**Results:**

Significant predictors were age, tumor stage, distant metastases, gamma-glutamyl transpeptidase (GGT) and alkaline phosphatase (ALP). The proportional-hazards model (nomogram) was based on pre-treatment characteristics. The nomogram had a concordance index (C-index) of 0.812 (95% CI 0.762–0.862), which was superior than the C-index of AJCC TNM Stage (0.755, 95% CI 0.702–0.808). The calibration plot in the validation cohort based on 5 predictors suggested good agreement between actual and nomogram-predicted OS probabilities. The decision curve showed that the nomogram in predicting OS is better than that of TNM staging system in all range. Moreover, patients were divided into three distinct risk groups for OS by the nomogram: low risk group, middle risk group and high risk group, respectively.

**Conclusion:**

This nomogram, using five pre-treatment characteristics, improves prediction of OS in patients with HBsAg-positive gastric cancer. It represents an improvement in prognostication over the current TNM stage. To generalize the use of this nomogram in other groups, additional validation with data from other institutions is required.

## Background

Gastric cancer (GC) is one of the most common malignancies and ranks the second leading cause of cancer death worldwide, with about one million new cases reported every year [1]. It was also commonly diagnosed and were recognized as leading causes of cancer death in China [1]. Chronic hepatitis B virus (HBV) infection has been well recognized as one of the major causes of hepatocellular carcinoma (HCC) [2]. However, in recent years, HBV infection has been reported to be associated with Gastric cancer (GC) [3], endometrial carcinoma [4] and nasopharyngeal carcinoma [5], though the underlying mechanism needs further investigation. Furthermore, HBV infection was associated with earlier cancer diagnosis and prognosis [6]. The biochemical parameters of liver function tests (LFT) are responsible for the metabolism and excretion of various endogenous and exogenous substances [7]. And for gastric cancer with HBsAg-positive, accurate assessment of liver function is key to the selection of treatment options.

The most commonly and widely used staging system for gastric cancer is the Union for International Cancer Control (UICC)/American Joint Committee on Cancer (AJCC) tumor, lymph node and metastases (TNM) staging system [8, 9]. The TNM staging system divides gastric cancer patients into different stages according to the depth of primary tumor invasion (T stage), regional lymph node metastasis (N stage) and distant metastasis (M stage) [10, 11]. Large variations are reported in the clinical outcomes, even patients with the same stage and similar treatment strategies [9, 12, 13]. This findings indicate that the present staging system is inadequate for predicting recurrence and does not reflect the biological heterogeneity of HBsAg-positive GC patients [12]. However, many other risk factors, such as age, sex and LFT should be considered for predicting individualized prognosis.

In this study, we aimed to develop and validate a prognostic nomogram that uses widely available pretreatment clinical and laboratory data to improve our ability to predict HBsAg-positive GC. We also performed a test to determine whether this model provides a more accurate prediction of prognosis when compared with TNM staging system.

## Methods

### Patient selection

The retrospectively study included 319 patients with histologically diagnosed GC with hepatitis B viral infection from 2009 to 2017 in Sun Yat-sen University Cancer Center (SYSUCC, Guangdong, China). All the patients were classified as the first record of hospitalizations and the clinical information were extracted from Electronic Medical Record (EMR) system. The levels of LFT factors were investigated before treatment Laboratory Information System (LIS). The inclusion criteria were as follows: (1) patients with a confirmed histologically diagnosed of GC; (2) patients with HBsAg-positive, but without other types of hepatitis viruses (i.e. hepatitis A viral, hepatitis C viral); (3) patients without second tumor, or indefinite diagnoses; (4) patients with complete clinical data; (5) patients without diseases influenced LFT (i.e. acute hepatitis, liver cirrhosis); (6) patients without any treatment. We divided patients into two cohorts by the time sequence. The primary cohort comprised 235 patients from August 2008 to September 2015. The validation cohort was contained 84 GC patients from September 2015 to January 2017 with age and sex match to the primary cohort. All patients provided written informed consent. The Institute Research Ethics Committee of the Sun Yat-Sen University Cancer Center, Guangzhou, China approved this study. The authenticity of this article has been validated by uploading the key raw data onto the Research Data Deposit public platform (http://www.researchdata.org.cn), with the approval RDD number as RDDA2019001020.

### Laboratory measurements

All the patients received routine tests at the first visit in our hospital. Blood samples were collected at room temperature, then centrifuged at 3500 r/min for 10 min, which could be used to estimate the level of LFT biomarkers, including alanine aminotransferase (ALT), aspartate aminotransferase (AST), lactate dehydrogenase (LDH), gamma-glutamyl transpeptidase (GGT), total bile acid (TBA), alkaline phosphatase (ALP), albumin (ALB), total bilirubin (TBIL), apolipoprotein A1 (ApoA1), apolipoprotein B (ApoB), prothrombin time (PT), fibrinogen (Fbg). HBV infection markers including HbsAg, hepatitis B surface antibody (HbsAb), hepatitis B e antigen (HbeAg) hepatitis B e antibody (HbeAb) and hepatitis B core antibody (HbcAb) were recorded.

### Follow-up

All GC patients were advised to receive regular follow-ups after completion of the primary therapy according to clinical guidelines. Patients were generally followed up every 3 months in the first 2 years and annually thereafter for patients without evidence of recurrence in the following 3 to 5 years. Patients who did not visit our hospital as scheduled were telephoned for follow-ups to obtain the treatment information and living status (performed by The Medical Information Unit in our Cancer Center). The last follow-up occurred in September 2018. The outcome of our study was overall survival (OS). OS was defined as the time from the diagnosis of HCC to the date of the last follow-up or death.

### Statistical analysis

Statistical analyses were performed using SPSS 16.0 (IBM, Chicago, IL, USA) and R for Windows (version 3.4.2, http://www.r-project.org/). The optimal cut-off points in our study were evaluated by minimum P value from log-rank × 2 statistics using the X-tile program [14] and continuous variables were transformed to categorical variables, while the categorical variables were classified based on clinical findings. Univariate and multivariate regression analysis was used to analyze the risk factors in the primary cohort, A nomogram was formulated based on the results of multivariate analysis by the package of rms. We tested the accuracy of the nomograms by discrimination and calibration both in primary and externa validation cohort. The discrimination of the nomogram was measured by Harrell’s C-index (C-index). The value of the C-index ranges from 0.5 to 1.0, with 0.5 indicating random chance and 1.0 indicating a perfect ability to correctly discriminate the outcome with the model. Then, the calibration curve of the nomogram model for the OS and decision curve analyses were performed. The total points of each patient were calculated according to the established Cox regression model, 3 groups of patients with different risk of prognosis (based on the total points) were delineated using the X-tile program. Survival curves were depicted by the Kaplan–Meier method, and using the dichotomized risk group as a factor, finally, compared using the log-rank test. All statistical tests were two-sided, and P values of less than 0.05 were considered to be statistically significant.

## Results

### Basic characteristics

The clinicopathologic characteristics of the training and validation sets were evaluated. The characteristics of the 235 consecutive AFP-negative HCC patients in the primary cohort and 84 patients in the validation cohort are showed in Table 1. There were 68 (28.94%) patients died, while 22 (26.19%) patients died respectively.

**Table 1:** Baseline clinical features

Characteristics	Development cohort (n = 235)	Validation cohort (n = 84)
	Mean ± SD/No (%)	Mean ± SD/No (%)
Age, year	54.95 ± 11.92	55.00 ± 11.58
Sex		
Male	163 (69.36%)	60 (71.43%)
Female	72 (30.64%)	24 (28.57%)
TNM stage		
I	43 (18.30%)	6 (7.14%)
II	66 (28.09%)	18 (21.43%)
III	83 (35.32%)	37 (44.05%)
IV	43 (18.30)	23 (27.38%)
Tumor stage		
T1	42 (17.87%)	6 (7.14%)
T2	25 (10.64%)	5 (5.95%)
T3	76 (32.34%)	29 (34.52%)
T4	92 (39.15%)	44 (52.38%)
Node stage		
N0	62 (26.38%)	15 (17.86%)
N1	41 (17.45%)	12 (14.29%)
N2	43 (18.30%)	17 (20.24%)
N3	89 (37.87%)	40 (47.62%)
Metastasis		
No	192 (81.70%)	61 (72.62%)
Yes	43 (18.30%)	23 (27.38%)
Location		
1	33 (14.04%)	14 (16.67%)
2	56 (23.83%)	23 (27.38%)
3	109 (46.38%)	32 (38.10%)
4	37 (15.74%)	15 (17.86%)
AST, U/L		
≤ 22.8	17.51 ± 2.66	17.65 ± 3.70
> 22.8	35.15 ± 21.05	44.82 ± 34.85
ALT, U/L		
≤ 30.1	16.67 ± 5.37	17.89 ± 5.94
> 30.1	56.41 ± 41.03	65.97 ± 47.55
LDH, U/L		
≤ 168.7	142.59 ± 16.72	146.24 ± 17.25
> 168.7	206.02 ± 54.78	289.90 ± 353.72
GGT, U/L		
≤ 26.9	16.52 ± 5.09	17.39 ± 5.02
> 26.9	51.02 ± 45.46	55.67 ± 57.97
TBA, μmol/L		
≤ 5.4	2.86 ± 1.25	3.21 ± 1.22
> 5.4	12.76 ± 10.41	13.10 ± 8.99
ALP, U/L		
≤ 80	61.72 ± 11.77	60.94 ± 10.51
> 80	98.59 ± 25.85	120.26 ± 83.86
ALB, g/L		
≤ 38.8	34.86 ± 3.74	36.16 ± 1.97
> 38.8	42.56 ± 2.42	43.03 ± 2.48
TBIL, μmol/L		
≤ 7.3	5.78 ± 1.26	5.98 ± 0.94
> 7.3	13.11 ± 4.77	12.40 ± 4.78
ApoA1, g/L		
≤ 1.1	0.96 ± 0.13	0.96 ± 0.12
> 1.1	1.34 ± 0.19	1.33 ± 0.21
ApoB, g/L		
≤ 1.1	0.84 ± 0.16	0.82 ± 0.16
> 1.1	1.44 ± 0.79	1.17 ± 0.04
PT, S		
≤ 11.9	11.11 ± 0.47	11.09 ± 0.52
> 11.9	12.56 ± 0.67	12.95 ± 1.66
Fbg, g/L		
≤ 3.6	2.63 ± 0.56	2.68 ± 0.54
> 3.6	4.35 ± 0.67	4.27 ± 0.70

Data are presented as mean (SD) or N (%)

### Biomarker selection

All the available informations, including clinicopathologic characteristics and biomarkers, were included for univariate and multivariate analysis (Table 2). In univariate analyse, Age, TNM stage, tumor stage, node stage, distant metastases, LDH, GGT, TBA, ALP, ALB, ApoB, Fbg were related to OS. All of the potentially important biomarkers identified in univariate analysis were further included in the multivariate analysis. Based on 235 HBsAg-positive GC patients with complete information, age, tumor stage, distant metastases, GGT and ALP were significant predictors of OS.

**Table 2:** Univariate and multivariate Cox hazards analysis between clinical features and OS

Characteristics	Univariate analysis		Multivariate analysis	
	HR (95% CI)	P-value	HR (95% CI)	P-value
Age, year	2.197 (1.177–4.101)	0.013	2.278 (1.172–4.428)	0.015
Sex				
Male/female	0.848 (0.503–1.429)	0.535		
TNM stage				
I/II/III/IV	3.135 (2.280–4.310)	< 0.001		
Tumor stage				
T1/T2/T3/T4	1.481 (1.074–2.043)	0.017	1.801 (1.293–2.510)	0.001
Node stage				
N0/N1/N2/N3	1.317 (1.019–1.701)	0.035		
Metastasis				
No/yes	4.753 (2.732–8.268)	< 0.001	5.164 (2.912–9.157)	< 0.001
Location				
1/2/3/4	0.958 (0.732–1.254)	0.757		
AST, U/L				
≤ 22.8/> 22.8	1.511 (0.934–2.446)	0.093		
ALT, U/L				
≤ 30.1/> 30.1	0.558 (0.267–1.168)	0.122		
LDH, U/L				
≤ 168.7/> 168.7	2.026 (1.252–3.281)	0.004		
GGT, U/L				
≤ 26.9/> 26.9	3.157 (1.929–5.167)	0.001	2.093 (1.223–3.581)	0.007
TBA, μmol/L				
≤ 5.4/> 5.4	2.065 (1.277–3.339)	0.003		
ALP, U/L				
≤ 80/> 80	3.393 (2.093–5.499)	< 0.001	2.161 (1.271–3.672)	0.004
ALB, g/L				
≤ 38.8/> 38.8	0.541 (0.316–0.837)	0.007		
TBIL, μmol/L				
≤ 7.3/> 7.3	0.623 (0.369–1.053)	0.077		
ApoA1, g/L				
≤ 1.1/> 1.1	0.564 (0.243–1.309)	0.182		
ApoB, g/L				
≤ 1.1/> 1.1	2.056 (1.101–3.841)	0.024		
PT, S				
≤ 11.9/> 11.9	1.464 (0.886–2.419)	0.137		
Fbg, g/L				
≤ 3.6/> 3.6	1.876 (1.052–3.346)	0.033		

### Development and validation of the prediction model

**Fig. 1:**
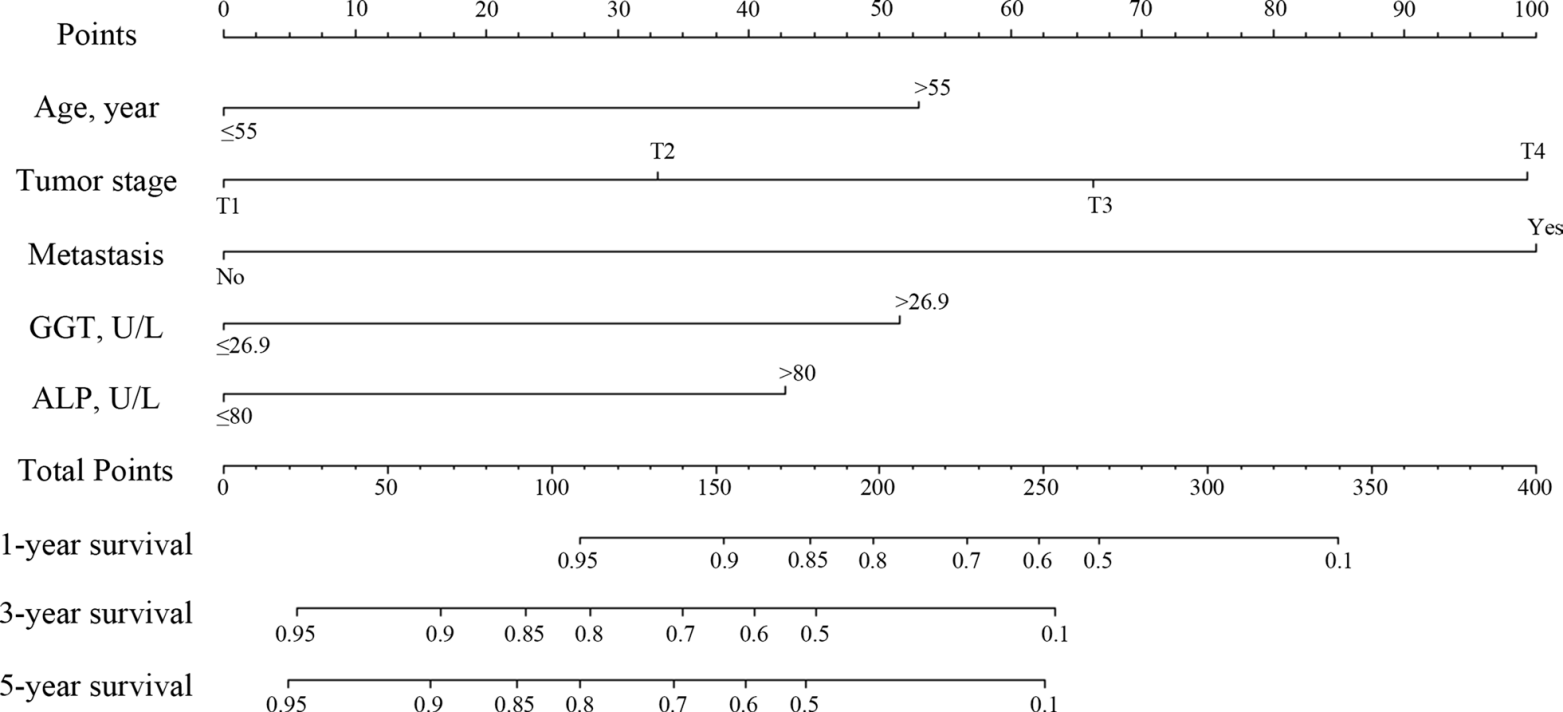
Nomogram, including age, tumor stage, distant metastases, GGT and ALP, for 1, 3 and 5 years overall survival (OS) in patients with HBsAg-positive GC. The nomogram is valued to obtain the probability of 1, 3 and 5 years survival by adding up the points identified on the points scale for each variable

**Fig. 2:**
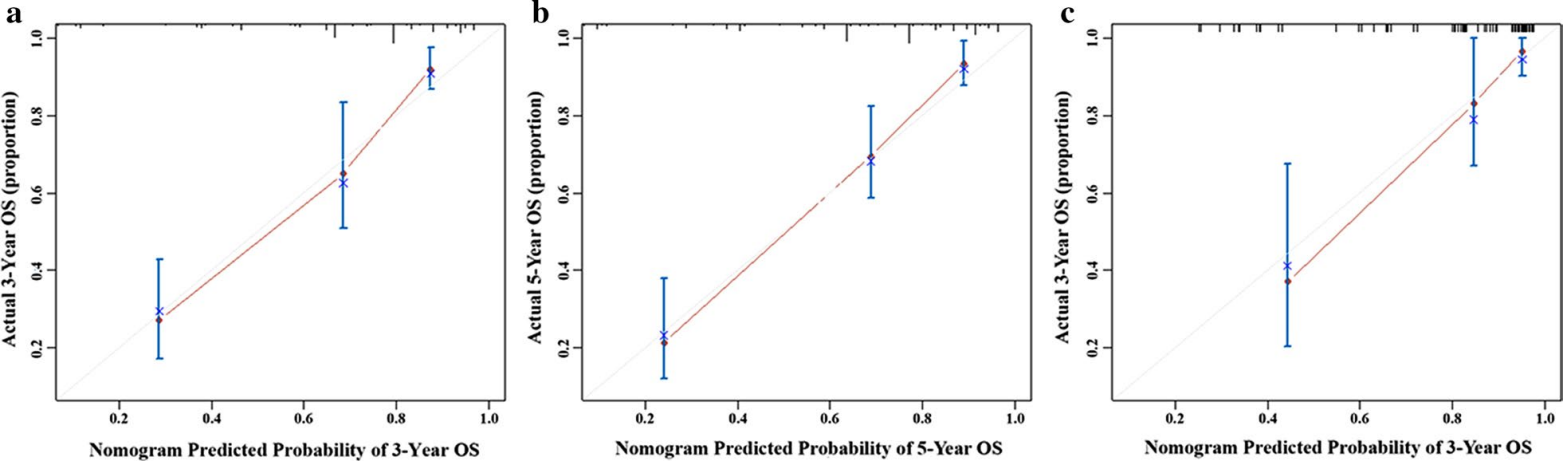
Calibration curve of the nomogram in the primary and validation cohort, with the x-axes are actual survival estimated by the nomogram, the y-axes are observed survival calculated by the Kaplan–Meier method. **a** Three-year OS in the primary cohort. **b** Five-year survival OS in the primary cohort. **c** Three-year OS in the validation cohort

A nomogram is a graphic representation of the solution of an equation that provides a reasonable approximation of the probability of a particular outcome. The model explanatory covariables consisted of age, tumor stage, distant metastases, GGT and ALP. A nomogram was constructed to predict 1-, 3- and 5-year OS (Fig. 1). The validation of nomogram was consisted of discrimination and calibration Discrimination was performed by using a concordance index (C-index) Calibration was evaluated by comparing the means of predicted survival with estimating of predicted with observed Kaplan–Meier survival, with the x-axes are actual survival estimated by the nomogram, the y-axes are observed survival calculated by the Kaplan–Meier method. In the primary cohort, the C-index for OS prediction was 0.812 (95% CI 0.762–0.862). The calibration plot for the probability of OS at 1, 3 or 5 year after therapy showed an optimal agreement between the prediction by nomogram and actual observation (Fig. 2).

### Validation of the predictive accuracy of nomograms for OS

We then applied the nomogram to the validation cohort of 84 HBsAg-positive GC patients.

The C-index for OS prediction was up to 0.821 (95% CI 0.723–0.919). The calibration plot for the probability of OS at 3-year after therapy showed an optimal agreement between the prediction by nomogram and actual observation (Fig. 2).

Furthermore, the discrimination of the nomogram and that of the AJCC TNM Stage have been compared. In the development cohort, the C-index of nomogram was 0.812 (95% CI 0.762–0.862), which was superior than the C-index of AJCC TNM Stage 0.755 (95% CI 0.702–0.808).

### Decision curve analysis

**Fig. 3:**
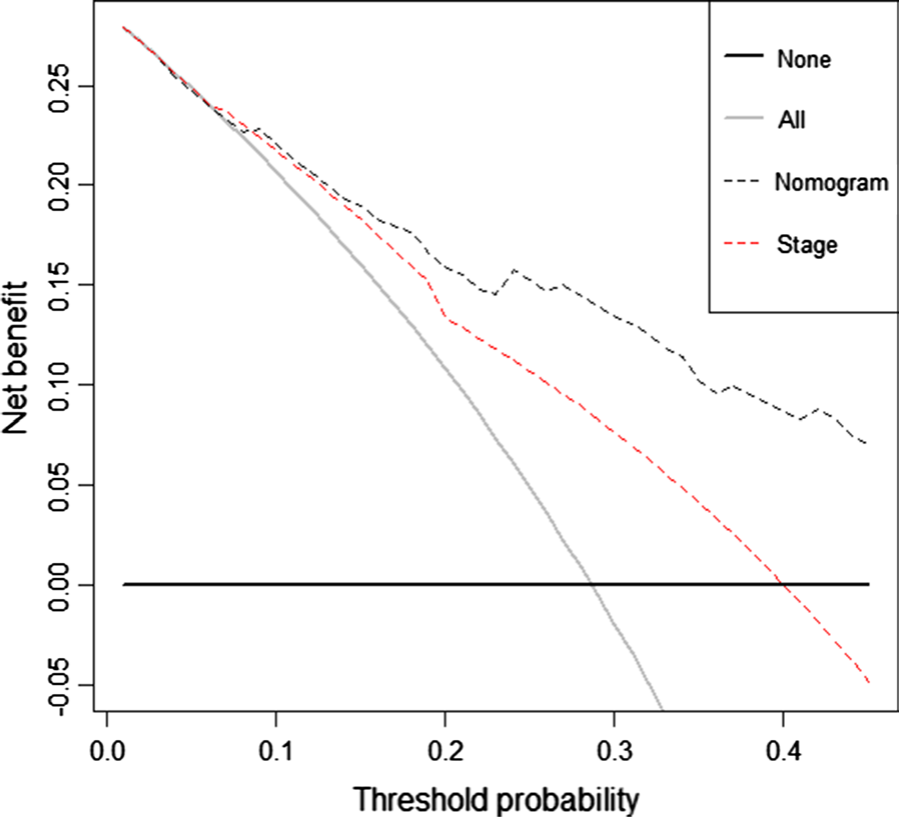
Decision curve analysis for overall survival. Black line: all patients dead. Gray line: none patients dead. Black dashed line: model of nomogram. Red dashed line: model of TNM staging system

The decision curve analysis for the nomogram and TNM staging systems is showed in Fig. 3. The decision curve presented that if the threshold probability of a patient is > 10%, the developed nomogram and TNM staging system in predicting OS is more benefit than all patients dead scheme or none patients dead scheme. Furthermore, the net benefit was comparable, the nomogram in predicting OS is more benefit than that of TNM staging system in this range.

### Comparison of predictive accuracy for OS between nomogram and TNM stage systems

**Fig. 4:**
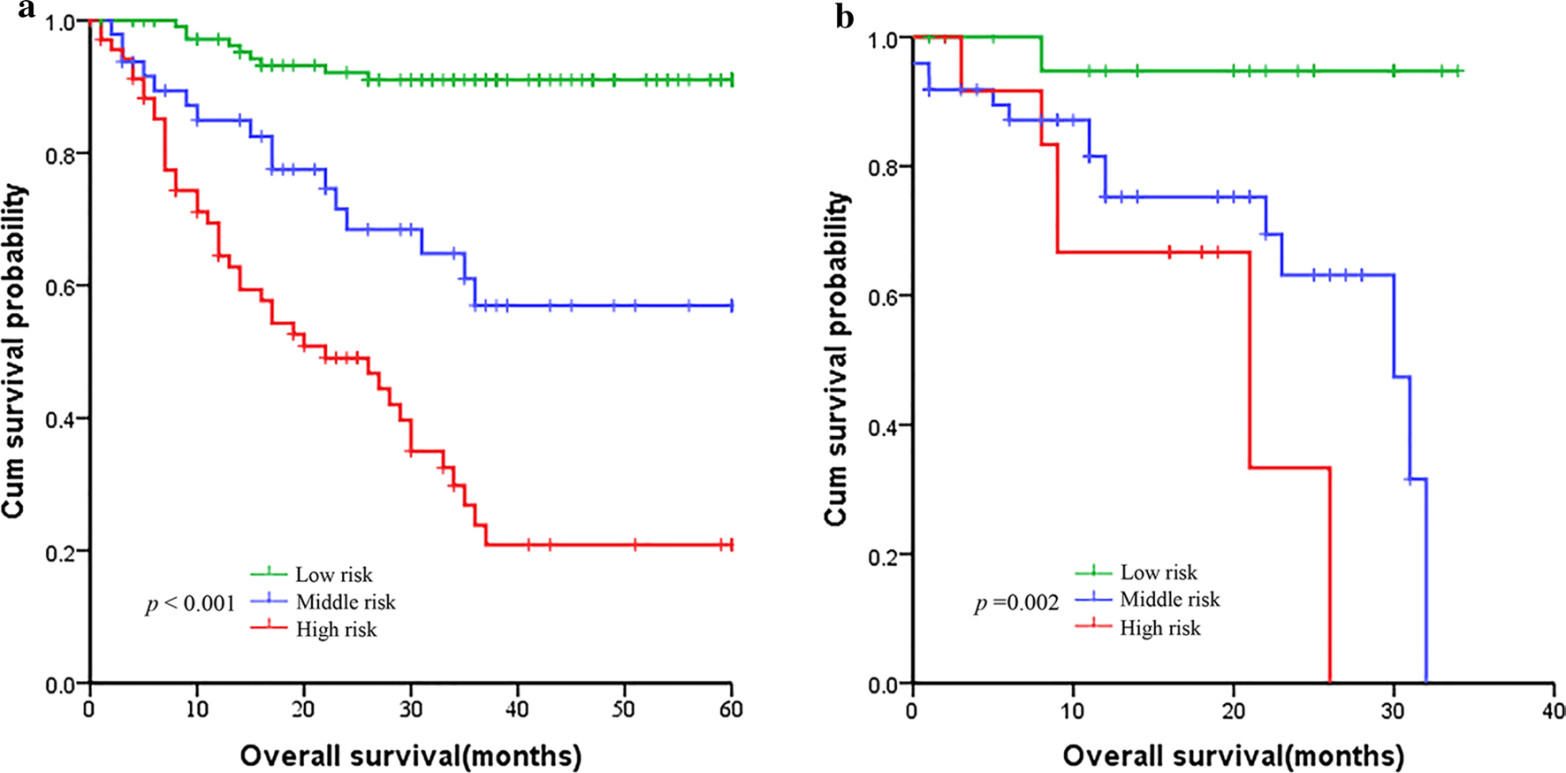
Kaplan-Meier survival curves of nomogram. **a** In the primary cohort. **b** In the validation cohort

Based on the nomogram, patients were divided into three groups: low-risk group, middle-risk group and a high-risk group, which showed good prognostic classification for HBsAg-positive GC both in development cohort and validation cohort. In the development cohort, there were 114 patients in the low-risk group, 49 patients in the middle-risk group, while 71 patients in the high-risk group. The OS between the three risk groups were (37.07 ± 16.80) months, (24.68 ± 18.31) months, (8.38 ± 6.33) months (P < 0.001). Also, in the validation cohort, there were 22 patients in the low-risk group, 49 patients in the middle-risk group, while 13 patients in the high-risk group. The mean OS between the three risk groups were (19.82 ± 10.07) months, (14.08 ± 9.76) months and (13.92 ± 7.15) months (P < 0.001) (Fig. 4).

## Discussion

Gastric cancer (GC) is one of the most common malignant diseases in the digestive system, contributing to about 10% of annual deaths from cancer [15, 16]. The accurate tumor prognosis after definitive treatment is indispensable. The prevalence of hepatitis B virus (HBV) infection varies largely worldwide. China is one of the relatively high prevalence area [17]. HBsAg recognized as an independent risk factor for both liver cirrhosis and hepatocellular carcinoma [18]. It was also found that patients with liver cirrhosis had a high prevalence of gastric ulcers [19] and an increased risk of GC [20]. But, several studies have probed the existence of HBV in GC [3, 6] and HBV infection was associated with GC [3]. As HBV infection also exists in gastric mucosa epithelial cells, it may be possible that HBV infection increases the risk of GC in a similar mechanism of HBV-related hepatocellular carcinoma. Therefore, for HBsAg-positive GC, it is important to consider the influence of HBV.

Traditional TNM staging system be used to assess the prognosis of HBsAg-positive GC [10, 21], which exists some drawbacks. The system only considers the anatomical extent of the disease without considering the liver biofunctional heterogeneity of HBsAg-positive GC, which does not fully reflect the accurate prognosis. It could not provides an more accurate estimate of prognosis particularly in patients with incurable cancers. Hence, we developed a prognostic nomogram to predict OS and treatment strategies guidance in HBsAg-positive GC with by using widely available baseline clinical and laboratory information. In our study, we found that tumor stage, age, distant metastases, GGT and ALP were the factors that influenced prognosis of patients according to the multivariate analysis. Patients with an earlier stage of T staging, M staging, and TNM stage, and a lower GGT and ALP level have improved survival rates. Not only should we consider the impact of HBV on liver function, but comprehensive liver function and TNM staging system, thus more accurate prediction of the patient’s prognosis.

Our nomogram is available to combine all these putative prognostic predictors into a summary measure for prediction of HBsAg-positive GC. It has demonstrated that the nomogram is better able to discriminate than the TNM staging system when used in GC patients with HBsAg-positive: In our model, the C-index for OS prediction was 0.812 (95% CI 0.762–0.862), for TNM staging system the C-index for OS prediction was 0.755 (95% CI 0.702–0.808). The nomogram showed better predictive accuracy for OS in development cohort. Simultaneously, validation of the nomogram also shows good predictive OS function. Furthermore, both in development cohorts and validation cohort, patients were divided into three group based on the nomogram, which could effectively discriminate the survival outcomes.

This nomogram is also a useful tool that utilizes conveniently available clinical information to provide simple prognostic information for oncologists and patients from complex statistical analysis. However, a major problem is to provide an accurate estimate of prognosis, especially, in patients with incurable cancers [22]. Traditional TNM staging system, which be used to assess the prognosis of GC, only considers the anatomical extent of the disease without considering the tumor heterogeneity. Previous articles have reported that the Italian Research Group for Gastric Cancer (GIRCG) prognostic scoring system (PSS) predicts the likelihood of recurrence after radical surgical treatment for GC, which is more accurate than TNM system to predict recurrence for high-risk patients [23]. Compared with the traditional TNM staging system, our method is more accurate and has a higher coincidence rate for patients with HBsAg-positive GC. Our method combines the clinical liver biochemical parameters with the TNM stage, taking into account anatomy and basic liver biochemical conditions, and more accurately predicts patients 1-OS, 3-OS, and 5-OS. Simultaneously, the decision curve showed that the nomogram in predicting OS is better than that of TNM staging system in all range.

There are also some shortcomings in our research. First, the nomogram was created based on data obtained from only one institution in China, lacking multi-center research data. Second, more patients are needed in the primary and validation cohorts. Finally, in the validation cohort, the follow-up time was shorter, and patients in the validation cohort still needed close monitoring and 5-year follow-up data. In addition, future research can incorporate the HBsAg-positive patient’s quality of life into the research system, and the nutritional status and quality of life of HBsAg-positive patients during the survival period have the same important status as the prolonged survival time. Despite these limitations, this nomogram represents a prognostic effect on patients with HBsAg-positive GC. We anticipate that this nomogram will stimulate ongoing research that will lead to improvements and access to a larger number of effective methods of prediction becomes available.

## Conclusions

In conclusion, we have developed a robust prognostic nomogram to predict the 3- and 5-year OS in patients with HBsAg-positive GC. This nomogram represents an improvement in prognostication over the current TNM stage. The proposed nomogram in this study provided statistically significantly better discrimination than the current TNM stage. To generalize the use of this nomogram in other groups, additional validation with data from other institutions is required.

## Abbreviations

GC: gastric cancer
LFT: liver function tests
ALT: alanine aminotransferase
AST: aspartate aminotransferase
LDH: lactate dehydrogenase
GGT: gamma-glutamyl transpeptidase
TBA: total bile acid
ALP: alkaline phosphatase
ALB: albumin
TBIL: total bilirubin
ApoA1: apolipoprotein A1
ApoB: apolipoprotein B
PT: prothrombin time
Fbg: fibrinogen
HBV: hepatitis B virus
HR: hazard ratio
95% CI: 95% confidence interval
OS: overall survival
